# B cells and energy metabolism in HER2-positive DCIS: insights into breast cancer progression from spatial-omics analyses

**DOI:** 10.1186/s13058-025-01990-2

**Published:** 2025-03-21

**Authors:** Helga Bergholtz, Jens Henrik Norum, Tonje Gulbrandsen Lien, Martina Landschoof Skrede, Øystein Garred, Therese Sørlie

**Affiliations:** 1 Department of Cancer Genetics, Institute for Cancer Research, Oslo University Hospital, Oslo, Norway; 2 Department of Pathology, Oslo University Hospital, Oslo, Norway; 3 Institute of Clinical Medicine, Faculty of Medicine, University of Oslo, Oslo, Norway

**Keywords:** DCIS, Breast cancer progression, Spatial-omics, Immune microenvironment, B cells, Energy metabolism

## Abstract

During breast tumor progression, the transition from ductal carcinoma in situ (DCIS) to invasive breast cancer is a critical step with large implications for prognosis. However, the mechanisms of invasion are still largely unknown. At the DCIS stage, there is an over-representation of HER2-positive lesions compared with invasive breast cancer. In this study, we investigated the associations between gene expression profiles in cancer cells and the immune microenvironment of HER2-positive DCIS and invasive breast tumors with concurrent DCIS using spatial transcriptomics. We found distinctly more B cells in the vicinity of DCIS ducts than in invasive tumor areas. There was higher expression of genes involved in energy metabolism in DCIS cancer cells than in invasive cancer cells and a positive correlation between expression of metabolic genes and B-cell abundance in DCIS. In contrast were processes related to epithelial to mesenchymal transition negatively correlated with B-cell abundance in DCIS. We also found significant correlation between expression of the B-cell-attracting chemokines CCL19, CCL21 and CXCL13 in stromal cells and B cell abundance in DCIS. This study indicates that B cells may play a protective role in the progression of HER2-positive DCIS to invasive breast cancer and that increased metabolic activity in intraductal cancer cells in combination with chemokines produced by stromal cells may influence the immune microenvironment of DCIS. These findings have implications for understanding HER2-positive breast cancer progression.

**Supplementary Information:**

The online version contains supplementary material available at 10.1186/s13058-025-01990-2.

## Background

In ductal carcinoma in situ (DCIS), cancer cells are confined within the mammary gland ducts. As such, these lesions are indolent and are considered clinically manageable with excellent prognoses [1, 2]. DCIS is generally accepted as a non-obligate precursor to invasive breast cancer [3]. Since the implementation of mammographic screening, the number of detected DCIS cases has increased, followed by an increase in the number of patients treated for DCIS [4, 5]. Most DCIS cases are treated surgically with or without adjuvant radiotherapy, resulting in very low recurrence rates [5]. However, if left untreated, many DCIS lesions never develop into invasive disease, indicating substantial overtreatment of patients diagnosed with DCIS [6, 7]. While there is much knowledge about prognostic and predictive biomarkers for invasive disease, there are no sufficiently good biomarkers for the invasive potential of DCIS [2].

The progression from DCIS to invasive ductal carcinoma (IDC) involves degradation of the basement membrane followed by invasion of cancer cells into surrounding tissue. This critical step may be initiated by the cancer cells, but a permissive tumor microenvironment is likely facilitating invasion, including changes in the myoepithelium and stromal cells [8–11]. Immune cells may play dual roles in the progression from DCIS to IDC, either by facilitating invasion or by detecting and destroying DCIS cancer cells that escape the mammary duct so that only cancer cells that outsmart the immune response are able to invade the stroma [11]. Additionally, mechanical forces and metabolic adaptations may contribute to invasion [12, 13].

The relevance of the intrinsic molecular subtypes in IDC is well known [14, 15], and several studies have explored these subtypes in DCIS [16–18]. An interesting observation from such studies is the distinct over-representation of HER2-positive DCIS compared with IDC, which contrasts the aggressive phenotype that HER2-positive tumors display in invasive disease [19, 20]. The reason for this apparent delayed invasion of HER2-positive DCIS is diverse and probably includes both cancer cell-intrinsic and -extrinsic factors. Immune cell recruitment to the surroundings of DCIS lesions may be of particular importance. Overall, HER2-positive DCIS has more tumor-infiltrating lymphocytes than does HER2-negative DCIS [21, 22]. Studies have shown that immune infiltrates in DCIS include a greater proportion of B cells than those in IDC, where T cells and macrophages dominate [23, 24]. B cell abundance is associated with HER2 positivity in DCIS, and B cells are often located in interductal immune cell aggregates [24, 25]. The reason for these variations in immune cell infiltration across DCIS subtypes is not known, but inherent molecular characteristics of intraductal cancer cells likely contribute to shaping the immune microenvironment and the attraction of different immune cells [26, 27].

In this study, we used spatial-omics methods to study HER2-positive pure DCIS and invasive breast tumors with concurrent DCIS. We mapped the degree of intratumoral heterogeneity, characterized the immune response and investigated the associations between gene expression in cancer cells and the stromal immune response. We detected a markedly greater abundance of B cells surrounding DCIS lesions, which correlated with the activation of specific metabolic pathways in intraductal cancer cells. These results may contribute to improving our understanding of the risk of invasion in HER2-positive breast cancer.

## Materials and methods

### Selection of cases

Formalin-fixed paraffin-embedded (FFPE) tissue blocks from grade 3 pure DCIS tumors and invasive breast tumors with concurrent DCIS (mixed invasive tumors) were obtained from the diagnostic biobank of Oslo University Hospital and from Istituto Nazionale dei Tumori, Milan, Italy. Histopathological diagnosis was confirmed by a pathologist using routine diagnostic criteria. Based on immunohistochemistry (IHC) and tumor morphology, eight HER2-positive cases (IHC 3+) harboring sufficient amount of tissue with intraductal cancer cell growth from each tumor type were selected, in total 16 cases. Tumors were not matched for any other clinicopathological factors. The samples were deidentified, and their use in research was approved by the institution’s internal review and ethics boards (approval numbers: 2016/433 (Oslo, Norway) and PG/U-25/01/2012-00001497 (Milan, Italy)).

### Immunohistochemistry

FFPE tissue was sectioned at 4 µm, and immunohistochemistry was performed according to a previously described protocol [28]. Antigen retrieval was performed using a steamer for 40 min in 10 mM sodium-citrate buffer (pH 6.0). The tissue was incubated with primary antibodies (anti-CD19, Cell Signaling, #90176, dilution 1:200, and anti-HER2, Roche, #790–4493, dilution 1:150) overnight before being incubated with secondary antibodies (Goat Anti-Rabbit IgG Antibody (H + L), Biotinylated, #BA-1000-1.5, dilution 1:25) and visualized with 3,3′-diaminobenzidine (DAB) substrate and hematoxylin counterstain.

### Spatial transcriptomics

Spatially resolved gene expression data were obtained using Nanostring’s GeoMx Digital Spatial Profiler with the Cancer Transcriptome Atlas assay [29–31]. This assay yields gene expression data for approximately 1800 genes and is designed to comprehensively profile tumor biology, including the tumor microenvironment and immune response. Digital spatial profiling was performed through Nanostring’s Technology Access Program in Seattle, USA, according to the vendor’s recommendations. Briefly, 4 µm tumor sections were placed on SuperFrost Plus slides with two cases per slide (eight slides in total). To identify relevant regions of interest (ROIs), we used three fluorescently labeled morphology markers: pancytokeratin (PanCK, Novus #NBP2-33200DL594, clone AE1/AE3, dilution 1:400) to detect epithelial (cancer) cells; CD45 (Cell Signaling Technology #13917BF, clone D9M8I, dilution 1:100) to detect immune cells; and smooth muscle actin (SMA, Invitrogen, #53–5760-82, clone 1A4, dilution 1:400) to detect myoepithelial cells, endothelial cells and myofibroblasts, in addition to the nuclear marker Syto-83 (dilution 1:25). The slides were baked for 2 h at 60 °C and deparaffinized before being subjected to antigen retrieval for 20 min with BOND Epitope Retrieval 2 (Leica Microsystems, #AR9640) and proteinase K treatment at 0.1 µg/ml for 15 min. The slides were incubated overnight with a high-plex mixture of oligo-linked probes and fluorescently labeled morphology markers.

### Segment selection

During slide preparation, one pure DCIS sample detached from the slide and was therefore excluded. Segment selection was performed using adjacent H&E-stained sections as guidance (Suppl. Figure 1). The cancer and stromal cell compartments were selected as separate segments, with PanCK as a segmentation marker, resulting in two segment types: PanCK-positive segments, which included epithelial (cancer) cells, and PanCK-negative segments, which included nonepithelial stroma. Owing to the different growth patterns of cancer cells in DCIS and invasive tumors, the ROI selection strategy differed between areas with intraductal growth (DCIS) and those with invasive cancer cells (Suppl. Figure 2). For DCIS areas, cancer cell ROIs were placed inside ducts, and only PanCK-positive cancer cells were selected. Stromal DCIS ROIs were placed outside the ducts, avoiding the basement membrane, and only PanCK-negative cells were selected. In invasive areas, where stroma and cancer cells are interwoven, ROIs were segmented on the basis of PanCK status, creating one cancer cell segment and one stroma cell segment within each ROI. After ROI selection, the tissue within each segment was illuminated with UV light to separate the unique oligos from the probes, followed by the collection of oligos. Even though UV light-initiated release of probes is highly precise in the GeoMx technology [32], an *erosion* parameter of 2 µm was set to reduce any potential “bleed-over” between cancer cell and stromal segments [33]. In the following, *segments* refer to the unit of cells subjected to sequencing. Segments from pure DCIS lesions are termed DCIS_pure_, whereas DCIS segments from mixed invasive tumors are termed DCIS_inv_.

### RNA sequencing

Library preparation and PCR were performed according to the manufacturer’s instructions, and the samples were purified twice using AMPure XP Beads (Beckman Coulter) at a 1.2X bead-to-sample ratio. Paired-end sequencing was performed using NextSeq6000 sequencer with an S4 v1.5 35-cycle flow cell (Illumina).

### Quality control and normalization

To avoid the potential influence of distinctly different expression patterns between cancer and stromal cells on data normalization, quality control and normalization were performed separately for cancer and stromal cells. In addition, a merged dataset including both cancer cell and stromal segments was generated for use in silico cell type deconvolution. Quality control and normalization were performed in Nanostring’s GeoMX software according to the vendor’s recommendations: Probes identified as global outliers were removed before the geometrical mean of the probes was calculated to obtain one value per gene. Next, target (gene) filtering was performed, excluding targets expressed above the limit of quantitation in fewer than 10% of the segments, followed by segment filtering, which excluded segments with fewer than 10% of targets above the limit of quantitation. Two stromal segments were removed in this step. The final cancer cell dataset included all 55 original segments and gene expression data for 1162 genes, whereas the final stroma dataset included 36 segments and gene expression data for 1585 genes (Suppl. Figure 3). Normalization was performed using the Q3 method according to current recommendations [34].

### Validation dataset

For validation, we used a published dataset including bulk tissue samples from 57 DCIS and 313 IDC for which gene expression data were obtained using Agilent Sureprint G3 Human Gene Expression 8 × 60 K microarrays (#G4851A) (Agilent Technologies, Santa Clara, USA) [16]. The cutoff between HER2-high and HER2-low samples was set to log_2_(*ERBB2*-expression) = 11, which ensured clear separation of the HER2-high and HER2-low groups in both DCIS and IDC.

### Data analysis

Data analyses were performed using R (version 4.4.0) in RStudio (version 2024.04.01) [35, 36]. Visualizations were made using *ggplot2* (version 3.5.1) and *Complex Heatmap* (version 2.20.0) [37, 38]. Hierarchical clustering was performed using *Euclidean* distance metric and *complete linkage* clustering. Principal component analyses, Pearson correlation, T tests, Kruskal Wallis tests and *χ*2 tests were performed using the *stats* package (version 4.4.0) [35]. Differential gene expression analyses of spatial data were performed to compare DCIS_inv_ and invasive cancer cell segments. To account for multiple segments from the same case, we used mixed models with Sample-ID as a random effect. For each gene, a model was fitted using the *lme4* package (version 1.1.35.3) with the formula *Gene Expression ~Tumor Type* + *(1|SampleID),* and P values were obtained using Satterthwaite’s degrees of freedom method using the *lmerTest* package (version 3.1.3) [39, 40]. No preselection of variables was performed prior to differential gene expression analyses since the results were used in subsequent gene set enrichment analyses (GSEA). False Discovery Rate (FDR) was used to correct for multiple testing. GSEA was performed using the *fgsea* package (version 1.30.0) with the hallmark gene set collection from the Molecular Signatures Database (MSigDB) [41–43]. Single-sample GSEA was performed using the method described by Barbie et al. [44]. Visualization of the GSEA results in network diagrams was performed using *igraph* (version 2.0.3) [45]. Since GeoMx technology yields average gene expression within segments and not at the single-cell level, immune cell abundance was estimated by in silico deconvolution using the *SpatialDecon* package (version 1.14.0) with the embedded SafeTME cell profile matrix, and specifying which segments contain cancer cells [46, 47]. In silico cell deconvolution of the validation dataset was performed using CIBERSORTx [48] with the same cell profile matrix.

### CosMx spatial single-cell protein analyses

Two of the cases (ID-09: pure DCIS; ID-08: mixed invasive tumor) were selected for spatial proteomic analyses using Nanostring’s CosMx platform and the immuno-oncology 64-plex antibody panel. Within each case, 20 to 25 ROIs of size 0.51 mm × 0.51 mm were analyzed, obtaining single-cell protein expression data for all cells within the ROI, in total 57,069 cells. Sample processing, staining, imaging, and cell segmentation were conducted at Nanostring’s CX lab, Amsterdam, following the methodology outlined by the vendor [49]. In brief, 5 µm sections of FFPE tumor tissue were placed onto Bond Plus slides (Leica, #S21.2113.A) and dried overnight at 37 °C, vacuum sealed, and stored at 4 °C until analysis. Before slide preparation, the slides were baked at 60 °C for 3 h. For semiautomated processing of FFPE tissues, the Leica BOND RX system was used. The samples were deparaffinized and subjected to epitope retrieval (20 min in ER1 solution), followed by coverslip application, overnight antibody incubation, and cyclic protein readout on the Spatial Molecular Imager (SMI), in accordance with NanoString’s recommendations. Visualization markers for morphology and cell segmentation (DAPI, PanCK, CD45, CD3, and CD298/B2M) were integrated into the preparation process. Fiducials solution (200 nm fluorescent beads) was included at a concentration of 5 × 10e−5% for use in glass mapping and tissue identification in addition to providing as reference points throughout the data collection. Cell typing was performed using CELESTA [50].

## Results

### Low intratumoral heterogeneity in HER2-positive DCIS and mixed invasive breast tumors

**Fig. 1:**
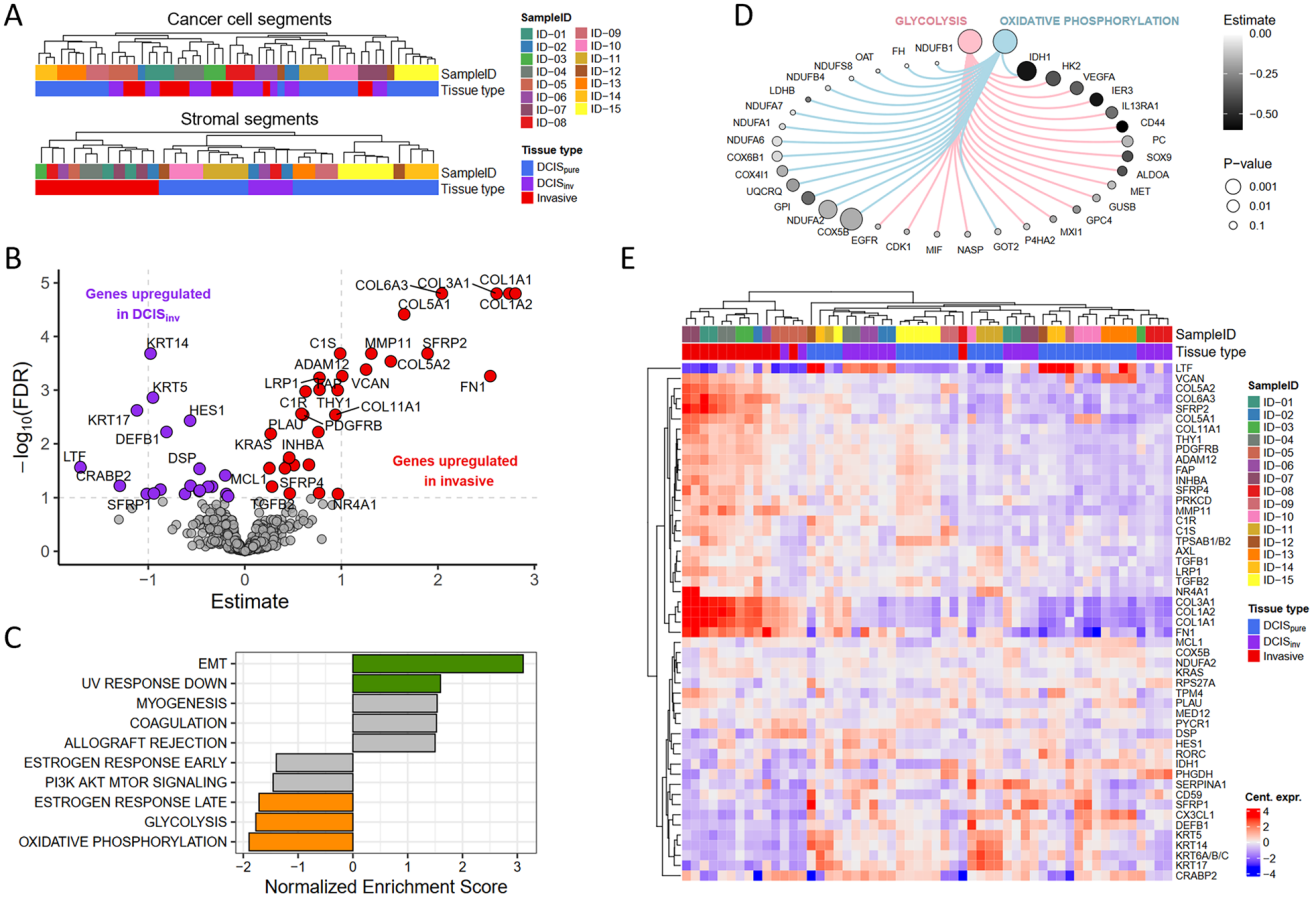
Genes and signatures distinguishing DCIS and invasive cancer cell segments. **a** *Hierarchical clustering dendrogram illustrating the relationship between cancer cell segments (top) and stromal segments (bottom).* Sample ID and tissue type is indicated by color. **b** *Differentially expressed genes between DCIS*_*inv*_* and invasive cancer cell segments using mixed effect models.* The model’s estimate (effect size) is shown on the x-axis and -log_10_(FDR) is shown on the y-axis. Genes significantly upregulated in invasive segments (FDR < 0.1) are shown in red and genes significantly upregulated in DCIS_inv_ segments are shown in purple. **c **Hallmark gene sets enriched between invasive cancer cell segments and DCIS_*inv*_*.* Normalized Enrichment Scores are shown for the top five signatures higher expressed in invasive segments (NES > 0) and the top five signatures higher expressed in DCIS_inv_ (NES < 0). Colored bars indicate significant signatures (FDR < 0.1). **d **Network of leading-edge genes enriched in DCIS_*inv*_* cancer cell segments.* Leading edge genes from glycolysis and oxidative phosphorylation signatures upregulated in DCIS compared to IDC. Node size is correlated to the inverse of the p value from DGE, and gray shading according to the estimate from the mixed effect models. **e** *Significantly differentially expressed genes between DCIS*_*inv*_* and invasive cancer cell segments (FDR* < *0.1).* The heatmap represents gene expression values centered across segments and hierarchically clustered across genes and segments (distance method: euclidean, clustering method: complete). Top annotation indicates sample ID and tissue type

To explore overall intra- and intertumoral heterogeneity, we performed hierarchical clustering based on gene expression of all genes, separately for cancer cell and stromal segments. Cancer cell segments from the same patient clustered together, indicating lower *intra*tumoral heterogeneity than *inter*tumoral heterogeneity (Fig. 1a). Additionally, cancer cell segments from the same mixed invasive tumors clustered together, regardless of whether they originated from invasive areas or DCIS. For the stromal data, segments grouped primarily according to tissue type and second according to sample ID (Fig. 1a). The low intratumoral heterogeneity was also evident from principal component analysis (Suppl. Figure 4A and 4B).

### Increased energy metabolism and downregulation of extracellular matrix remodeling genes in HER2-positive DCIS

We next compared gene expression between cancer cell segments from DCIS and invasive areas from the mixed tumors (Fig. 1b, Suppl. Table 1), followed by GSEA using the Hallmark signatures (Fig. 1c, Suppl. Table 1). In invasive segments, the top enriched gene set was related to epithelial-to-mesenchymal transition (EMT). The genes that contributed the most to this enrichment were not canonical EMT genes, but rather genes encoding components of the extracellular matrix (ECM), such as collagens, *VCAN*, and *FN1*, and genes involved in ECM degradation such as *MMP11*, *ADAM12*, and *FAP*. Importantly, these genes were expressed at lower levels in both DCIS_inv_ and DCIS_pure_ compared to invasive cancer cell segments (Suppl. Figure 5). Two metabolic signatures *Glycolysis* and *Oxidative Phosphorylation* were enriched in DCIS_inv_ cancer cell segments compared with invasive cancer cell segments, indicating higher metabolic activity in DCIS (Fig. 1c). Network analyses revealed little overlap between genes driving the enrichment of these two signatures, indicating that both processes were indeed more active in DCIS cells than in invasive cancer cells (Fig. 1d). Hierarchical clustering of the significantly differentially expressed genes (FDR < 0.1, n = 51) across all cancer cell segments revealed that most DCIS_pure_ segments clustered together with DCIS_inv_, highlighting similarities between DCIS cancer cells from pure and mixed tumors that were not captured by the initial hierarchical clustering (Fig. 1e).

### Different immune cell abundances, distributions and compositions in DCIS and IDC

**Fig. 2:**
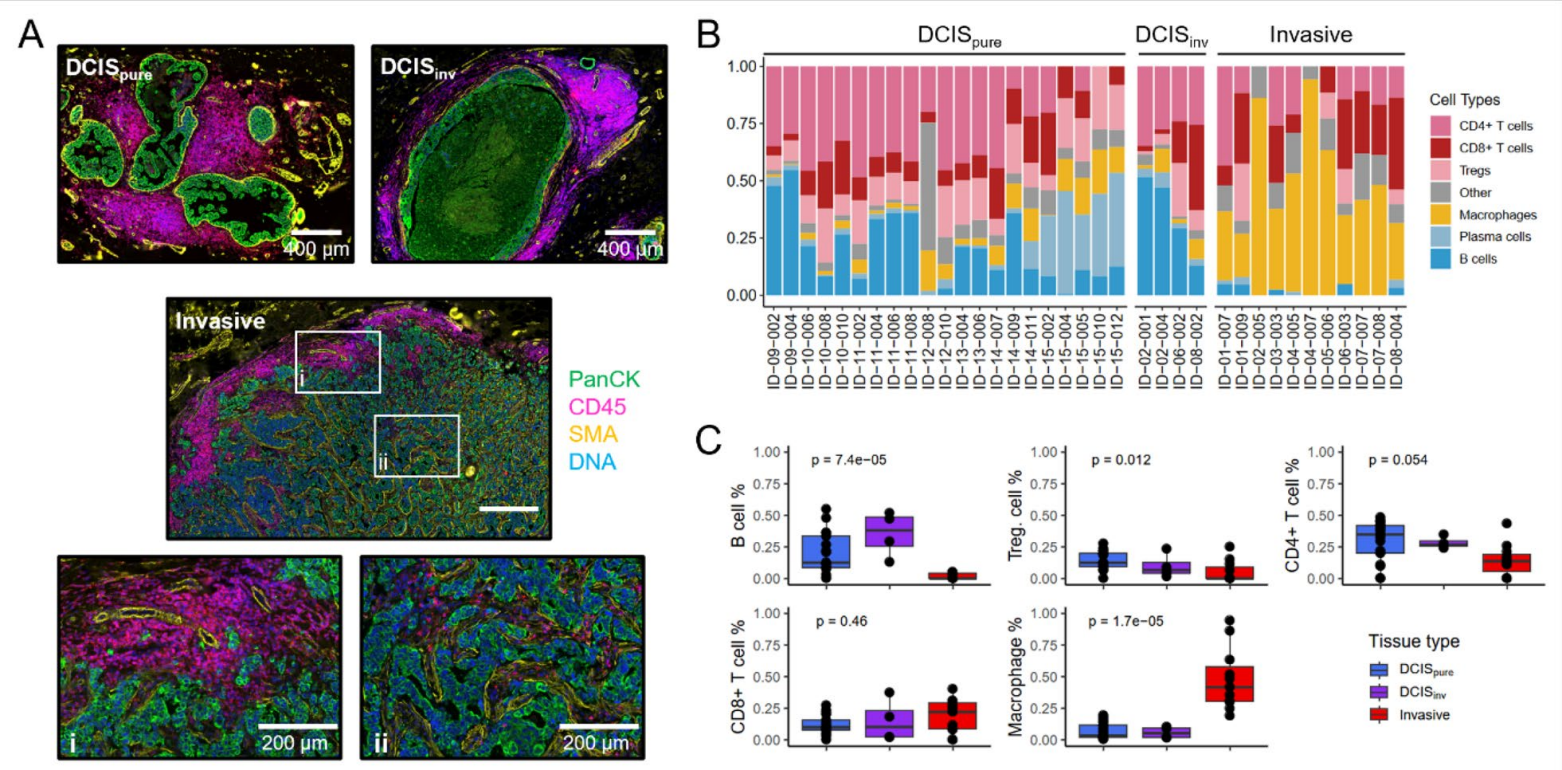
Immune cell profiling. **a** *Immune cell distribution in pure DCIS and mixed invasive tumors.* Immunofluorescence images of a pure DCIS (top left) and an area with DCIS from a mixed invasive tumor (top right). Shown below is an invasive area from a mixed invasive tumor and magnified images of an area from the periphery (i) and a central area (ii). PanCK (green), SMA (yellow), CD45 (magenta), Syto83 (DNA, blue). **b** In silico *immune cell deconvolution of stromal segments.* Each bar represents one stromal segment. The height of the colored bars represents the relative estimated abundance of the different immune cells. **c** *Abundance of immune cell types.* The percentage of estimated abundance of five selected immune cell types are shown on the y-axis and tissue type is shown on the x-axis. Boxplots illustrate the median (middle line) and the third and first quartiles (box); whiskers indicate 1.5 × IQR above and below the box. *P* values are from Kruskal Wallis tests

The immune cell abundance varied substantially between the patients. DCIS lesions in pure and mixed tumors were commonly surrounded by numerous immune cells, often organized as distinct aggregates, particularly in close proximity to DCIS ducts. In areas with invasive tumor growth, immune cells typically accumulated along the edges of the tumor, while they were sparse in the center of the tumor and, if present, frequently located along connective tissue fibers (Fig. 2a). In silico cell deconvolution of the stromal segments revealed greater estimated immune cell abundances in DCIS than in invasive tumors (Suppl. Figure 6A). Furthermore, the estimated immune cell composition also differed considerably between DCIS and invasive stromal segments (Fig. 2b). Interestingly, the immune profile of stromal segments from DCIS_inv_ resembled DCIS_pure_ rather than corresponding invasive segments from the same mixed invasive tumor, confirming the results from the initial hierarchical clustering (Fig. 1a). Compared with invasive segments, DCIS from pure and mixed invasive tumors presented a greater estimated abundance of B cells, in addition to more T regulatory and CD4^+^ T cells (the latter marginally non-significant), whereas stromal segments from invasive areas presented a greater abundance of macrophages (Fig. 2c). CD8^+^ T cells were not significantly differently abundant between the tissue types. The expression of the B cell marker genes *CD19*, *CD79A* and *MS4A1* (CD20) highly correlated with the estimated B cell abundance (Suppl. Figure 6B). IHC with an anti-CD19 antibody confirmed the different abundances of B cells between DCIS and mixed invasive tumors (Suppl. Figure 6C).

### Immune cell composition validated using single-cell spatial proteomics

**Fig. 3:**
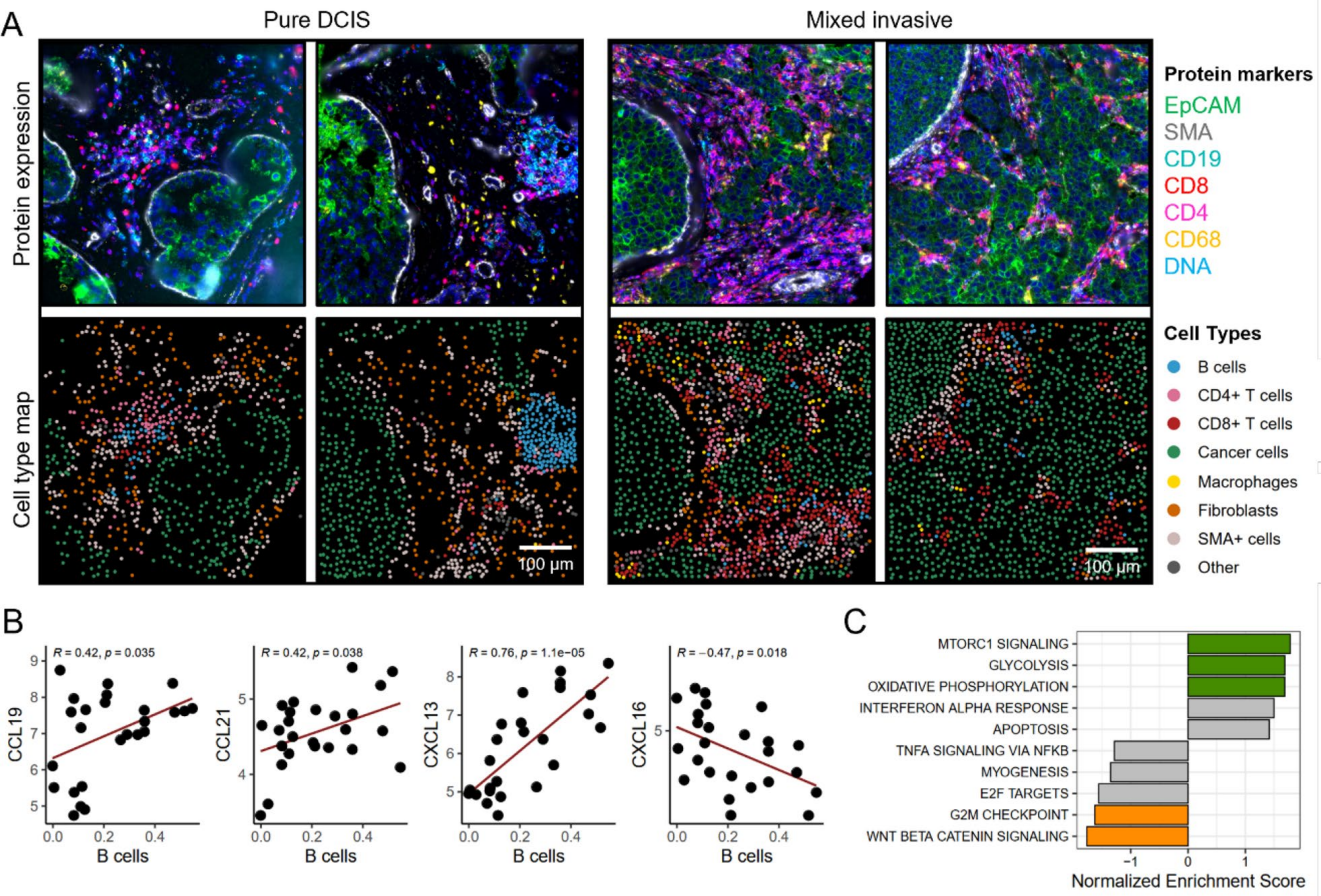
Single-cell analyses and immune cell signaling. **a** *Single-cell proteomic analyses of one pure DCIS and one mixed invasive tumor.* Two selected ROIs from each sample are shown. Left panels: ID-09 (pure DCIS), right panels: ID-08 (mixed invasive tumor)*.* Top: Composite immunofluorescence images of selected markers. EpCam (green), SMA (gray), CD19 (cyan), CD8 (red), CD4 (magenta), CD68 (yellow), DAPI (DNA, blue). Bottom: Maps of cell types estimated by CELESTA cell phenotyping indicated by colors for the different cell types. **b** *Correlation between chemokine gene expression and B cell abundance in stromal segments surrounding DCIS.* B-cell abundance estimated using immune cell deconvolution is shown on the x-axis and chemokine expression in stromal segments on a log_2_ scale is shown on the y-axis. Pearson correlation coefficients and corresponding *P* values are given. **c** *Gene Set Enrichment of genes expressed in DCIS cancer segments correlating with B cell abundance in corresponding stromal segments.* Normalized Enrichment Score is shown for the top five signatures positively correlated with B cell abundance (NES > 0) and the top five signatures negatively correlated with B cell abundance (NES < 0). Colored bars indicate significant signatures (FDR < 0.1)

To map the spatial distribution of single immune cells and to validate the immune cell deconvolution analysis, we performed single-cell spatial proteomic data analysis and immune cell phenotyping on two of the cases, one mixed invasive tumor (ID-08) and one pure DCIS (ID-09). In total 45 ROIs were analyzed obtaining single-cell data from 57,069 cells. The immune cells in the pure DCIS sample were predominantly B cells and CD4^+^ T cells, with few CD8^+^ T cells and macrophages (Suppl. Figure 6D). In contrast, mixed invasive tumors were dominated by CD8^+^ T cells, with a markedly lower abundance of B cells than pure DCIS tumors (Fig. 3a, Suppl. Figure 6D). Macrophages were heterogeneously distributed and most abundant in areas with invasive tumor growth. In both cases, B cells were commonly located in proximity to DCIS foci, and in the pure DCIS case, B cells were frequently present in distinct lymphoid aggregates. In addition to numerous B cells, these aggregates commonly included CD4^+^ T cells, dendritic cells and endothelial cells, a cell composition commonly observed in tertiary lymphoid structures (TLSs). Few T regulatory cells were observed, which contrasts the results from the immune cell deconvolution estimations. Apart from this, the single-cell spatial proteomic analyses of the two cases confirmed the results from the in silico deconvolution.

### B cell abundance in DCIS is associated with stromal expression of chemokines and high metabolic activity in cancer cells

Accumulation of B cells around DCIS foci suggests a localized signaling mechanism that recruits specific immune cells. Using gene expression data from the stromal segments, we examined the relationship between chemokine expression and B cell abundance as estimated by immune cell deconvolution. This revealed a positive correlation between B cell abundance and the expression of several B-cell-attracting chemokines in stromal segments surrounding DCIS, notably *CCL19*, *CCL21*, and *CXCL13* (Fig. 3b and Suppl. Table 2). These chemokines were highly expressed in the stromal regions of both DCIS types and lower expressed in invasive stromal segments (Suppl. Figure 6E). Conversely, *CXCL16* exhibited a negative correlation with B cell abundance and was expressed at low levels in DCIS, and higher in invasive stroma (Fig. 3b, Suppl. Figure 6E).

To explore which molecular pathways in DCIS cancer cells are associated with B cell abundance, we correlated gene expression values from cancer cell segments with the estimated B cell abundance in corresponding stromal segments, followed by GSEA (Fig. 3c, Suppl. Table 3). This analysis showed that WNT/β-catenin signaling and G2M checkpoint signatures in DCIS cancer cells were negatively associated with B cell abundance. In contrast, DCIS cancer cell segments surrounded by high number of B cells showed enrichment for signatures related to MTORC1 signaling, oxidative phosphorylation, and glycolysis. This suggests a link between elevated metabolic activity in DCIS cancer cells and increased B cell presence. These findings align with our previous differential gene expression analyses comparing DCIS and invasive cancer cell segments (Fig. 1c), with substantial overlap in the leading-edge genes from both these analyses (Suppl. Figure 6F and Fig. 1d). Notably, isocitrate dehydrogenase 1 (*IDH1*), the top hit, showed a significant positive correlation with B-cell abundance in DCIS (Suppl. Figure 6G) and higher expression in DCIS compared to invasive tumors (Suppl. Figure 6H).

### Bulk tissue analyses confirmed different B cell abundances in DCIS and invasive tumors

**Fig. 4:**
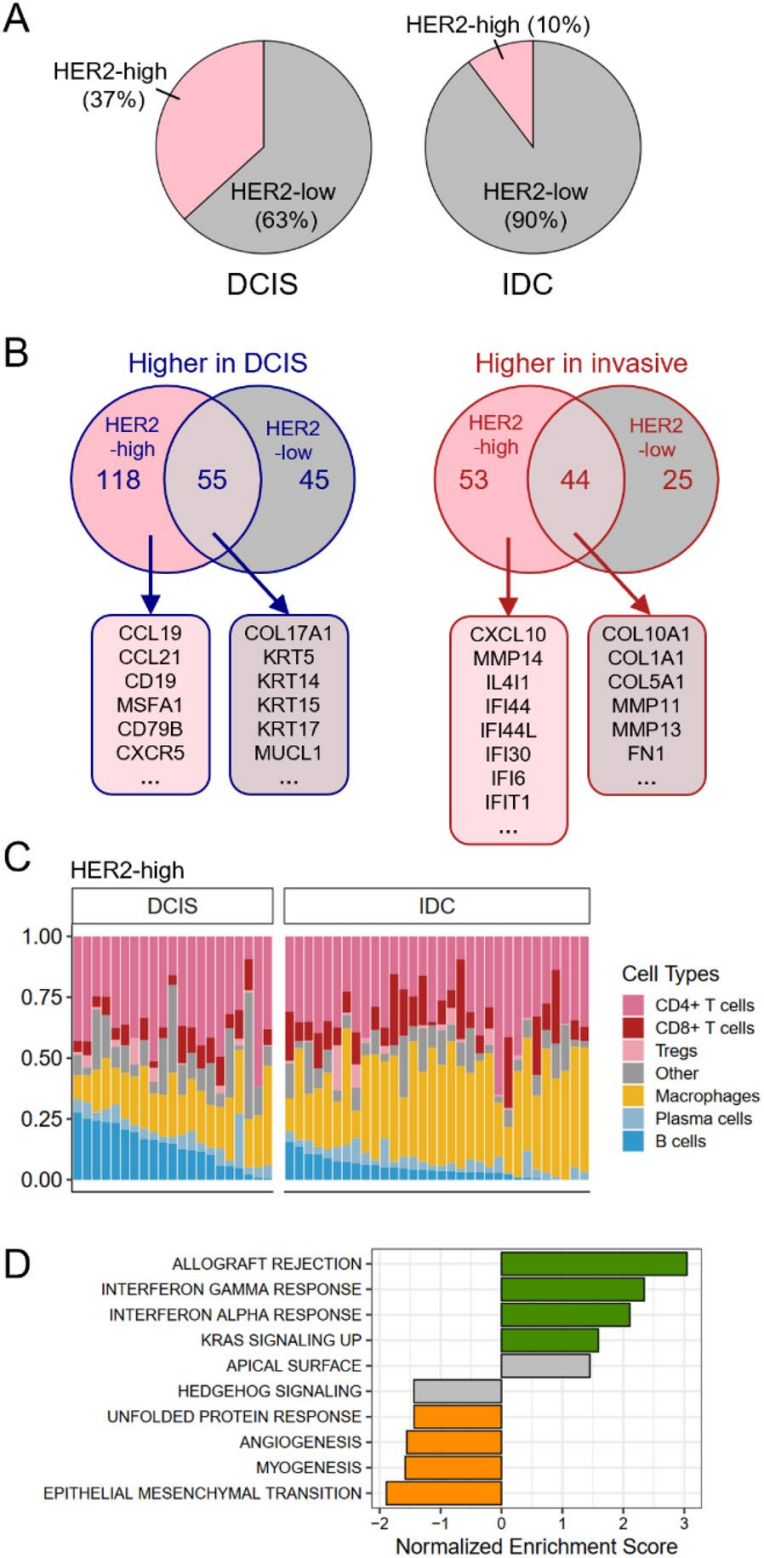
Gene expression and cell type abundance in an independent cohort. **a** *Distribution of HER2-high breast tumors (n* = *53) vs HER2-low (n* = *317) in DCIS and IDC.* HER2-high samples were defined as having log_2_(HER2-expression) > 11. The pink area indicates proportion of HER2-high tumors, and the gray area indicates HER2-low. Chi square test indicated significantly different distribution of HER2-high tumors between DCIS and IDC (*X*^2^ (df = 1) = 25,71, *p* < 0.001). **b** *Comparison of differentially expressed genes in HER2-high and HER2-low groups.* Numbers indicate significantly differentially expressed genes in each group (FDR < 0.05). Selected genes are shown in corresponding boxes. **c** *Immune cell deconvolution in HER2-high DCIS and invasive tumors.* Each bar represents one sample, and the height of the colored bars represent the relative estimated abundance of different immune cells. **d** *Top enriched Hallmark gene sets correlated with B-cell abundance in HER2-high DCIS.* Normalized Enrichment Scores for the top five signatures positively correlated with B-cell abundance (NES > 0) and the top five signatures negatively correlated with B-cell abundance (NES < 0) in HER2-high DCIS. Colored bars indicate significant signatures (FDR < 0.1)

To validate the findings from the spatial transcriptomic analyses in a larger dataset and to compare the profiles of HER2-positive tumors with those of HER2-negative tumors, we explored a previously published gene expression dataset derived from bulk tissue samples, including DCIS and invasive tumors, across all subtypes [16]. We divided the cohort into *HER2-high* (n = 53) and *HER2-low* (n = 317) based on *ERBB2* expression*.* As expected, the proportion of HER2-high tumors was significantly greater (*X*^2^ (df = 1) = 25,71, *p* < 0.001) among DCIS patients than among invasive patients (Fig. 4a). We performed DGE analyses to compare HER2-high DCIS and HER2-high invasive tumors, followed by GSEA (Suppl. Table 4). HER2-high invasive tumors were characterized by upregulation of signatures involving interferon response, EMT and cell cycle, whereas HER2-high DCIS tumors exhibited upregulation of numerous molecular pathways, including several involved in cell metabolism (Suppl. Figure 7A, Suppl. Table 4). Next, we performed DGE analysis between HER2-low DCIS and IDC, followed by a comparison of the significantly differentially expressed genes from the two DGE analyses (Fig. 4b). HER2-high DCIS showed upregulation of several immune markers and chemokines, including many of those identified in the spatial transcriptomic analyses, such as *CCL19*, *CCL21*, *CD19* and *MSF4A1*, whereas HER2-high IDC showed higher expression of several interferon-induced genes (Fig. 4b, Suppl. Table 4). Similar to the spatial data, *IDH1* expression was significantly greater in DCIS tumors than in invasive tumors in both the HER2-high and HER2-low groups (Suppl. Figure 7B).

To explore the immune cell composition of DCIS and invasive tumors in both the HER2-high and HER2-low groups, we performed in silico immune cell deconvolution (Fig. 4c and Suppl. Figure 7C). We detected a lower abundance of macrophages and a greater abundance of B cells and CD4 + T cells in HER2-high DCIS tumors than in HER2-high invasive tumors, which is in good accordance with the results from the spatial transcriptomic analyses. The estimated abundance of B cells correlated strongly with the expression of the B cell markers *CD19, MS4A1* (CD20) and *CD79a* (Suppl. Figure 7D).

The expression of the chemokines *CCL19*, *CCL21* and *CXCL13* correlated significantly with B cell abundance in the HER2-high group in the validation dataset (Suppl. Figure 7E) and was greater in HER2-high DCIS than in HER2-high invasive tumors and all HER2-low tumors (Suppl. Figure 7F). *CXCL16* did not correlate with B-cells and showed no difference in expression between any of the groups. Furthermore, we correlated the estimated B cell abundance with gene expression followed by GSEA in HER2-high DCIS. As expected, multiple immune-related pathways were positively correlated with B cell abundance, whereas EMT was negatively correlated with B-cell abundance (Fig. 4d, Suppl. Table 5). We did not find enrichment of metabolic pathways, possibly due to contributions from noncancerous cells in the bulk tumor tissue masking more subtle differences.

## Discussion

In bulk tissue analyses, stromal and immune cells will, to a variable degree, influence gene expression data obtained from a tumor. This complicates interpretation of the relationship between cell types, their spatial location, and gene expression. To resolve this challenge, spatial-omics technologies have emerged as invaluable tools for studying smaller areas of cell populations separately [33]. Spatial-omics is particularly useful for studying DCIS because of its unique morphology, with cancer cells located inside mammary gland ducts. In this study, we explored gene expression profiles in HER2-positive pure DCIS and mixed invasive breast carcinomas in a spatial context with the aim of identifying clues to how HER2-positive DCIS progresses to invasive disease.

Overall, we found high resemblance between cancer cell segments obtained within the same tumor specimen and between DCIS foci and invasive cancer cell segments within mixed invasive tumors. This intrinsic resemblance is in accordance with previous literature reporting few consistent molecular differences between DCIS and invasive areas in mixed tumors [51–53]. In the stroma, there was high concordance of overall gene expression and estimated immune cell composition within the same patient; however, DCIS-associated stromal segments from mixed invasive tumors were remarkably similar to those obtained from pure DCIS.

Our results revealed a greater abundance of B cells in HER2-positive DCIS than in HER2-positive invasive tumors and HER2-negative DCIS and invasive tumors, validating previous findings [23, 24]. In addition, we detected increased B-cell abundance in DCIS areas within invasive tumors. Tumor-infiltrating B cells (TIL-Bs) have been shown to be a marker for favorable prognosis in several tumor types [54, 55]; however, B cells can also play a pro-tumorigenic role [56]. In DCIS, B cells may either act anti-tumorigenic by incapacitating invading cancer cells through a local humoral response and activation of cytotoxic T cells, or pro-tumorigenic by contributing to a permissive microenvironment for invasion. High TIL-Bs are prognostic of better outcomes in patients with HER2-positive IDC [57]. In DCIS, TIL-Bs have not been studied extensively; however, in one study by Miligy and colleagues, TIL-Bs were shown to be a marker for shorter recurrence-free survival [24]. The authors reported a significant association between TIL-Bs and HER2 status but did not study the role of B cells specifically in HER2-positive DCIS. Our study lacks information on recurrence; however, the observed higher B cell abundance in pure DCIS compared with IDC may indicate, in contrast to what was found in the study by Miligy et al., that B cells in HER2-positive DCIS play a protective role. In a study by Risom and colleagues, non-progressing DCIS were characterized by increased abundance of immune cells and increased desmoplasia combined with, and potentially as a response to, thinning of the myoepithelium [10]. We did not characterize the myoepithelium specifically in our study, however, the large immune cell influx seen in the DCIS cases indicates communication between intraductal cancer cells and the extraductal microenvironment.

In DCIS cases, B cells were commonly located in immune cell aggregates. In the pure DCIS case analyzed by single-cell proteomics, we observed that B cells in such aggregates colocalized with CD4 + T cells. These aggregates potentially represent tertiary lymphoid structures (TLSs), which are ectopic lymphoid organs that can form at sites of persistent inflammation [23–25]. Mature TLSs are considered important players in anti-tumorigenic immune responses, and their formation is stimulated by the chemokine CXCL13 [56]. *CXCL13* was indeed more highly expressed in the stromal compartment of DCIS, as assessed by the spatial transcriptomics analysis, and in the HER2-high group in the bulk gene expression validation dataset. The stromal expression of *CXCL13* was highly correlated with B cell abundance. Moreover, the chemokines *CCL19 *and *CCL21* were highly correlated with B cell abundance. These are known to play a role in the recruitment of immune cells to tissues [58]. Outside of lymph nodes, *CCL19* and *CXCL13* are commonly produced by dendritic cells and macrophages while *CCL21* is produced by cells in the high-endothelial venules [58]. In the bulk validation data, we found higher expression of *CCL19*, *CCL21* and *CXCL13* in the HER2-high DCIS compared to HER2-high invasive tumors and compared to both HER2-low groups. These findings indicate a unique immune response in HER2-positive DCIS, potentially holding clues to the overrepresentation of HER2-positive DCIS.

HER2 overexpression in invasive breast cancer has been linked to increased metabolic activity, including upregulation of the mTOR pathway and increased glycolysis [59]. In DCIS, the role of metabolic activity is not established. Risom et al. showed that glycolysis was associated with non-progressing DCIS [10], while in a study by Strand et al., glycolysis and oxidative phosphorylation was associated with progression [60]. In our study, cancer cell segments from DCIS patients presented high expression of genes involved in energy metabolism compared to invasive cancer cell segments, and glycolysis and oxidative phosphorylation pathways were upregulated in DCIS. Interestingly, both these metabolic processes correlated with B cell abundance in DCIS. Increased metabolic activity in DCIS cancer cells may occur as a result of the highly restrictive and hypoxic DCIS environment [13]. One can speculate that this increased metabolic activity of DCIS cancer cells may influence the influx of immune cells (including B cells) towards the ducts. One potential mechanism involves intermediate metabolites such as α-ketoglutarate. α-ketoglutarate is produced in the TCA cycle from isocitrate by IDH1 and has been shown to stimulate anti-tumor immunity [61]. *IDH1* was indeed more highly expressed in DCIS than in IDC in our study and has also previously been shown to decrease with the progression of breast cancer [62].

WNT/β-catenin and cell cycle (G2M) signatures, which are associated with tumor aggressiveness, were negatively correlated with B cell abundance. EMT-related signatures were enriched in invasive tumors compared to DCIS in both spatial and bulk data, while negatively correlated to B cell abundance in DCIS, indicating that DCIS with a mesenchymal phenotype, attract less B cells. Hence, it is tempting to speculate that HER2-positive DCIS with few associated B cells and high expression of ECM-modulating proteins are more likely to progress than DCIS with the opposite phenotype. These findings again support a protective role of B cells in HER2-positive DCIS, as has also been hypothesized by others [26].

Spatial transcriptomics optimized for FFPE tumor tissue is a powerful tool for delineating cellular function and pathological changes and enables the use of routine pathology samples, utilizing very little tissue. Our study included a small number of cases; however, the study was limited to HER2-positive tumors, thus reducing the intertumoral heterogeneity that enables sufficiently reliable comparisons between groups. Follow-up data were not available for the patients in our study. A further limitation is the underlying different growth patterns of DCIS and invasive cancer cells, which instigates a segment selection strategy that could result in differences in the influence of stroma on the gene expression of the two types of cancer cell segments. However, this issue was reduced in our study by setting an appropriate erosion measure between the different segment types prior to probe collection. Finally, by using PanCK as a segmentation marker, we risk detecting signals in stroma from cancer cells with low or no cytokeratin expression. We have attempted to reduce the impact of such contamination in the immune cell deconvolution analysis by including tumor cell signatures.

Our study illustrates the usefulness of spatial omics, particularly for DCIS, because of its unique morphology and high degree of heterogeneity. Using these techniques, we found indications of a protective B cell immune response that may provide clues for understanding the biology of HER2-positive DCIS tumor progression.

## Supplementary Information

Additional file 1

Additional file 2

Additional file 3

Additional file 4

Additional file 5

Additional file 6

## Data Availability

Data from this study is available on https://figshare.com/projects/Data_included_in_Bergholtz_et_al_B_Cells_and_energy_metabolism_in_HER2-Positive_DCIS_Insights_into_breast_cancer_progression_from_spatial-omics_analyses_/239462. The validation dataset used in this study is described in the following metadata record: 10.6084/m9.figshare.12293102.

## Abbreviations

DAB: 3,3′-Diaminobenzidine
DCIS: Ductal carcinoma in situ
ECM: Extracellular matrix
EMT: Epithelial-to-mesenchymal transition
FDR: False discovery rate
FFPE: Formalin-fixed paraffin-embedded
GSEA: Gene set enrichment analyses
IDC: Invasive ductal carcinoma
IDH1: Isocitrate dehydrogenase 1
IHC: Immunohistochemistry
MSigDB: Molecular signatures database
PanCK: Pancytokeratin
ROI: Region of interest
SMA: Smooth muscle actin
SMI: Spatial molecular imager
TIL-B: Tumor-infiltrating B cell
TLS: Tertiary lymphoid structure
