# Supplementary material for: B cells and energy metabolism in HER2-positive DCIS: insights into breast cancer progression from spatial-omics analyses
Source: Breast Cancer Res. 2025 Mar 21;27:44. doi: 10.1186/s13058-025-01990-2 (PMC11929220; doi:10.1186/s13058-025-01990-2)
Supplement: Supplementary file 6 — Additional file 6 [file 13058_2025_1990_MOESM6_ESM.pptx]

## Slide 1
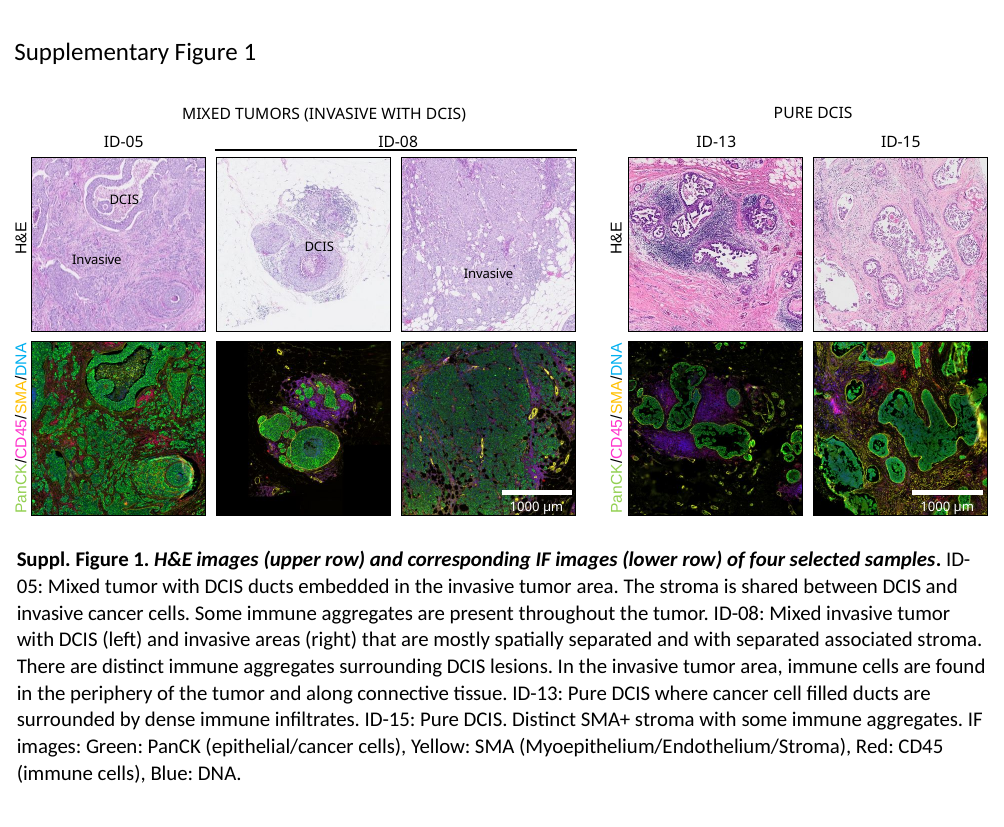

Supplementary Figure 1
PURE DCIS
MIXED TUMORS (INVASIVE WITH DCIS)
ID-05
ID-08
ID-13
ID-15
DCIS
H&E
H&E
DCIS
Invasive
Invasive
PanCK/CD45/SMA/DNA
PanCK/CD45/SMA/DNA
1000 µm
1000 µm
Suppl. Figure 1. H&E images (upper row) and corresponding IF images (lower row) of four selected samples. ID-05: Mixed tumor with DCIS ducts embedded in the invasive tumor area. The stroma is shared between DCIS and invasive cancer cells. Some immune aggregates are present throughout the tumor. ID-08: Mixed invasive tumor with DCIS (left) and invasive areas (right) that are mostly spatially separated and with separated associated stroma. There are distinct immune aggregates surrounding DCIS lesions. In the invasive tumor area, immune cells are found in the periphery of the tumor and along connective tissue. ID-13: Pure DCIS where cancer cell filled ducts are surrounded by dense immune infiltrates. ID-15: Pure DCIS. Distinct SMA+ stroma with some immune aggregates. IF images: Green: PanCK (epithelial/cancer cells), Yellow: SMA (Myoepithelium/Endothelium/Stroma), Red: CD45 (immune cells), Blue: DNA.

## Slide 2
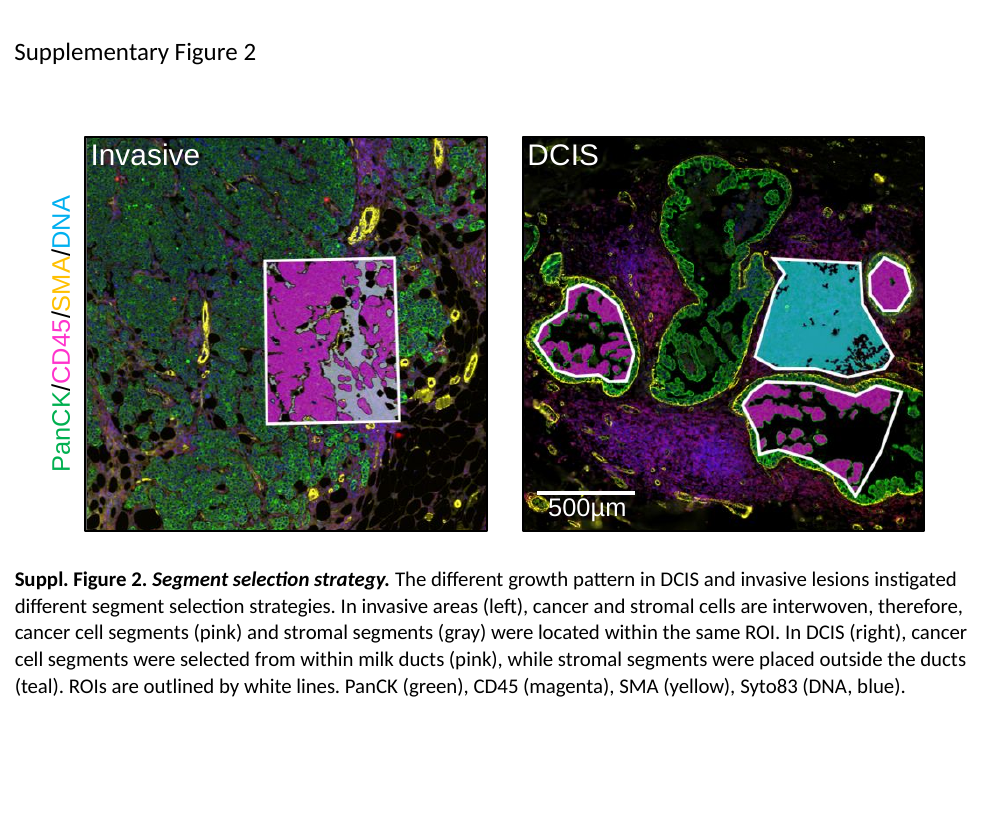

Supplementary Figure 2
DCIS
Invasive
PanCK/CD45/SMA/DNA
500µm
Suppl. Figure 2. Segment selection strategy. The different growth pattern in DCIS and invasive lesions instigated different segment selection strategies. In invasive areas (left), cancer and stromal cells are interwoven, therefore, cancer cell segments (pink) and stromal segments (gray) were located within the same ROI. In DCIS (right), cancer cell segments were selected from within milk ducts (pink), while stromal segments were placed outside the ducts (teal). ROIs are outlined by white lines. PanCK (green), CD45 (magenta), SMA (yellow), Syto83 (DNA, blue).

## Slide 3
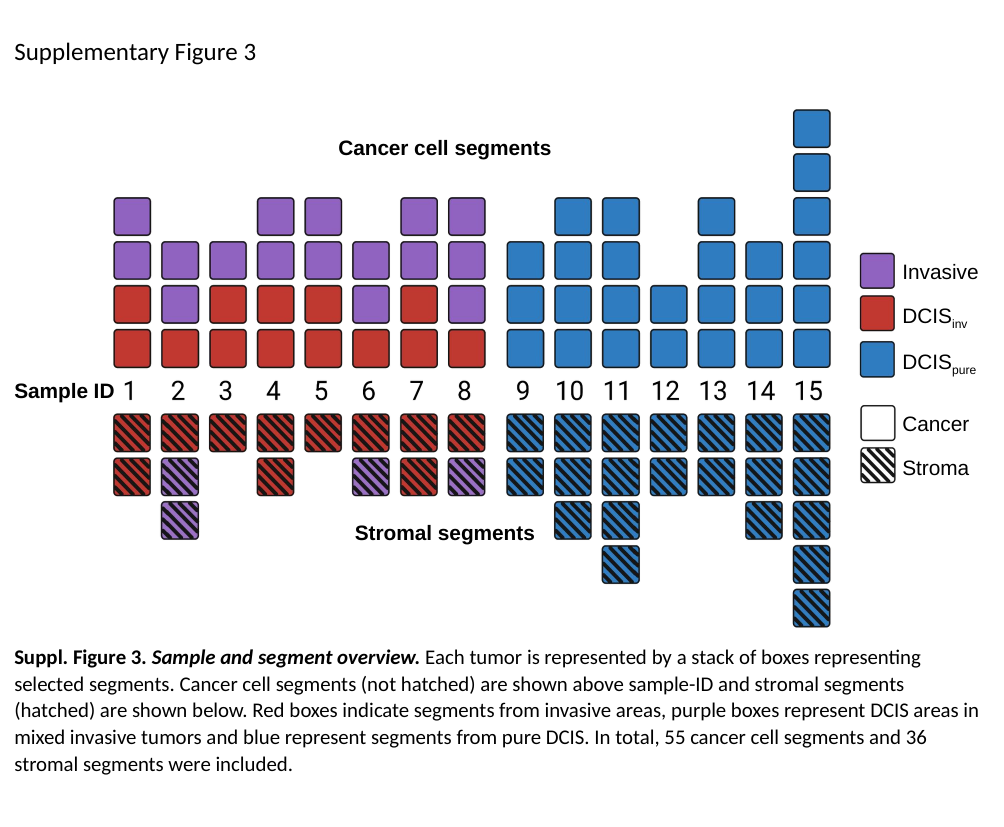

Supplementary Figure 3
Cancer cell segments
Invasive
DCISinv
DCISpure
Sample ID
Cancer
Stroma
Stromal segments
Suppl. Figure 3. Sample and segment overview. Each tumor is represented by a stack of boxes representing selected segments. Cancer cell segments (not hatched) are shown above sample-ID and stromal segments (hatched) are shown below. Red boxes indicate segments from invasive areas, purple boxes represent DCIS areas in mixed invasive tumors and blue represent segments from pure DCIS. In total, 55 cancer cell segments and 36 stromal segments were included.

## Slide 4
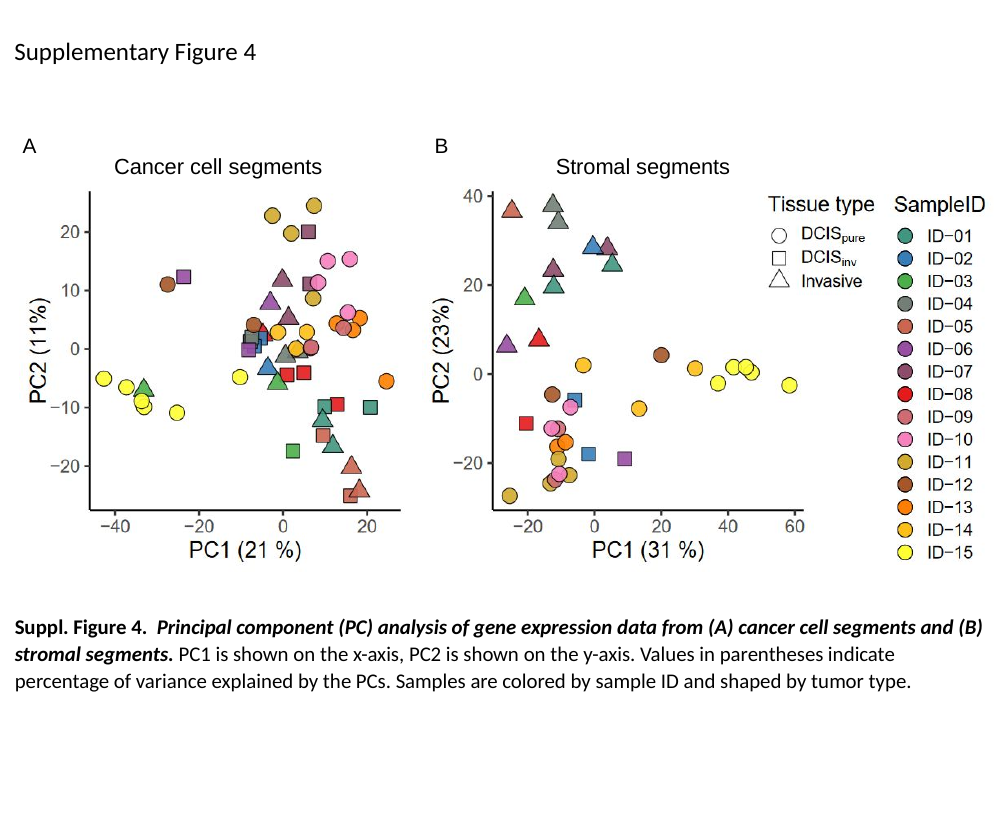

Supplementary Figure 4
A
B
Cancer cell segments
Stromal segments
Suppl. Figure 4. Principal component (PC) analysis of gene expression data from (A) cancer cell segments and (B) stromal segments. PC1 is shown on the x-axis, PC2 is shown on the y-axis. Values in parentheses indicate percentage of variance explained by the PCs. Samples are colored by sample ID and shaped by tumor type.

## Slide 5
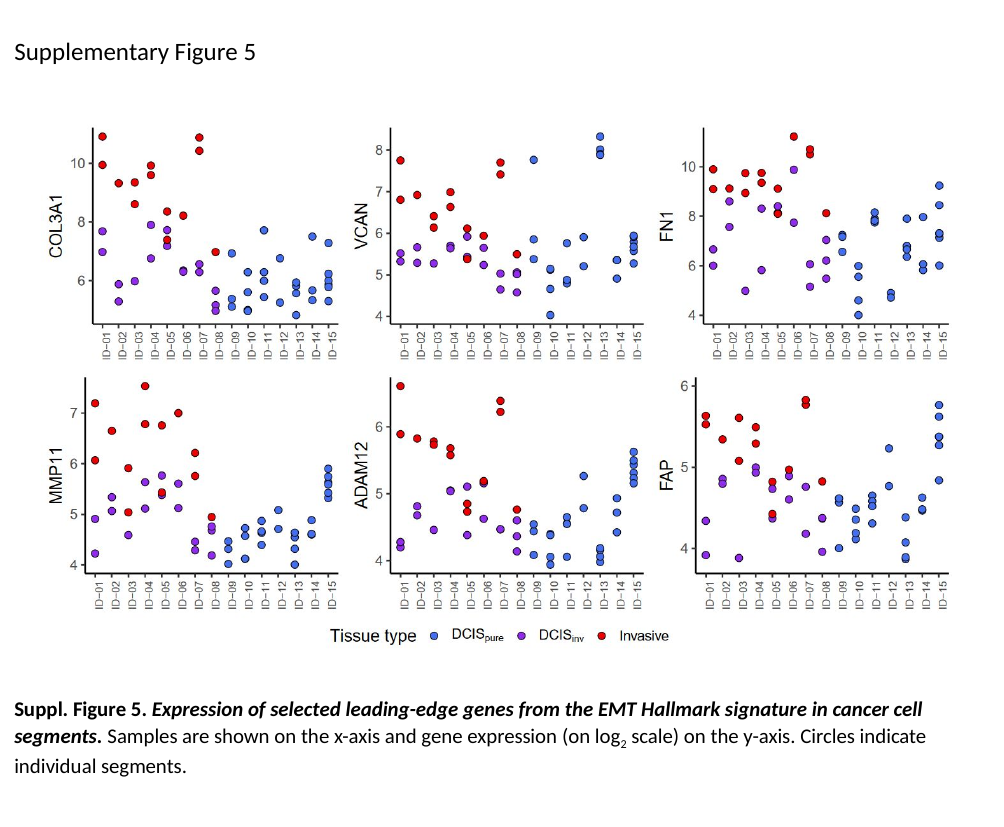

Supplementary Figure 5
Suppl. Figure 5. Expression of selected leading-edge genes from the EMT Hallmark signature in cancer cell segments. Samples are shown on the x-axis and gene expression (on log2 scale) on the y-axis. Circles indicate individual segments.

## Slide 6
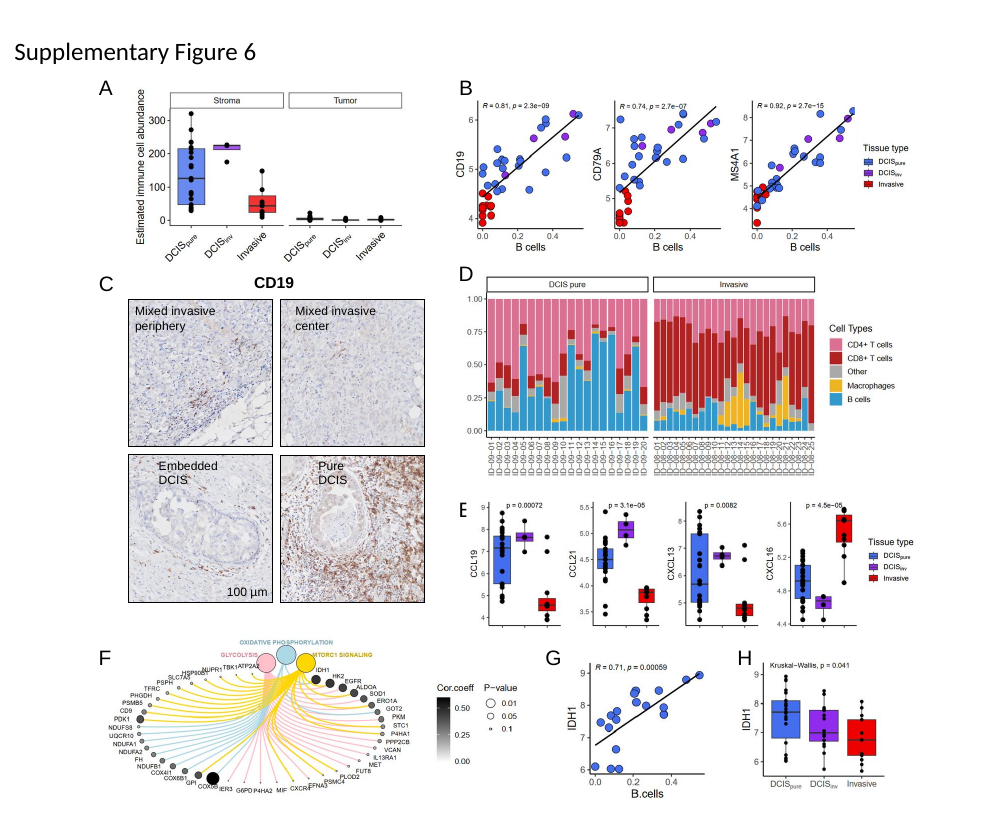

Supplementary Figure 6
B
A
D
C
CD19
Mixed invasive periphery
Mixed invasive center
100 µm
Embedded DCIS
Pure DCIS
E
F
G
H

## Slide 7
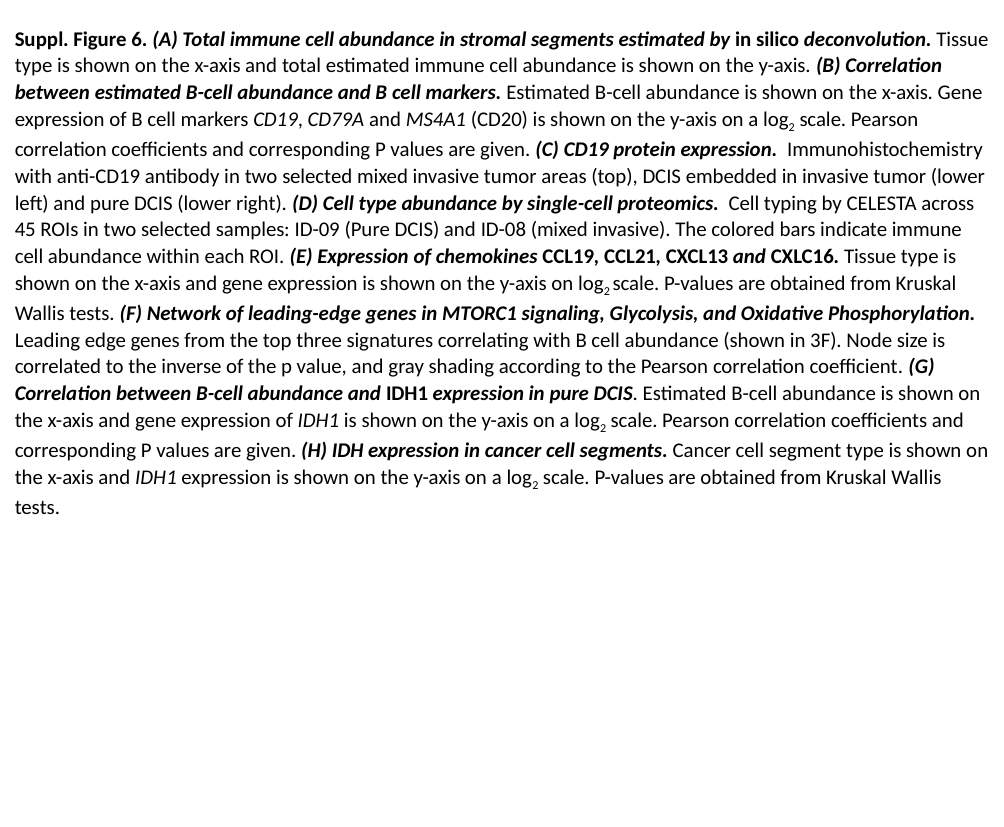

Suppl. Figure 6. (A) Total immune cell abundance in stromal segments estimated by in silico deconvolution. Tissue type is shown on the x-axis and total estimated immune cell abundance is shown on the y-axis. (B) Correlation between estimated B-cell abundance and B cell markers. Estimated B-cell abundance is shown on the x-axis. Gene expression of B cell markers CD19, CD79A and MS4A1 (CD20) is shown on the y-axis on a log2 scale. Pearson correlation coefficients and corresponding P values are given. (C) CD19 protein expression. Immunohistochemistry with anti-CD19 antibody in two selected mixed invasive tumor areas (top), DCIS embedded in invasive tumor (lower left) and pure DCIS (lower right). (D) Cell type abundance by single-cell proteomics. Cell typing by CELESTA across 45 ROIs in two selected samples: ID-09 (Pure DCIS) and ID-08 (mixed invasive). The colored bars indicate immune cell abundance within each ROI. (E) Expression of chemokines CCL19, CCL21, CXCL13 and CXLC16. Tissue type is shown on the x-axis and gene expression is shown on the y-axis on log2 scale. P-values are obtained from Kruskal Wallis tests. (F) Network of leading-edge genes in MTORC1 signaling, Glycolysis, and Oxidative Phosphorylation. Leading edge genes from the top three signatures correlating with B cell abundance (shown in 3F). Node size is correlated to the inverse of the p value, and gray shading according to the Pearson correlation coefficient. (G) Correlation between B-cell abundance and IDH1 expression in pure DCIS. Estimated B-cell abundance is shown on the x-axis and gene expression of IDH1 is shown on the y-axis on a log2 scale. Pearson correlation coefficients and corresponding P values are given. (H) IDH expression in cancer cell segments. Cancer cell segment type is shown on the x-axis and IDH1 expression is shown on the y-axis on a log2 scale. P-values are obtained from Kruskal Wallis tests.

## Slide 8
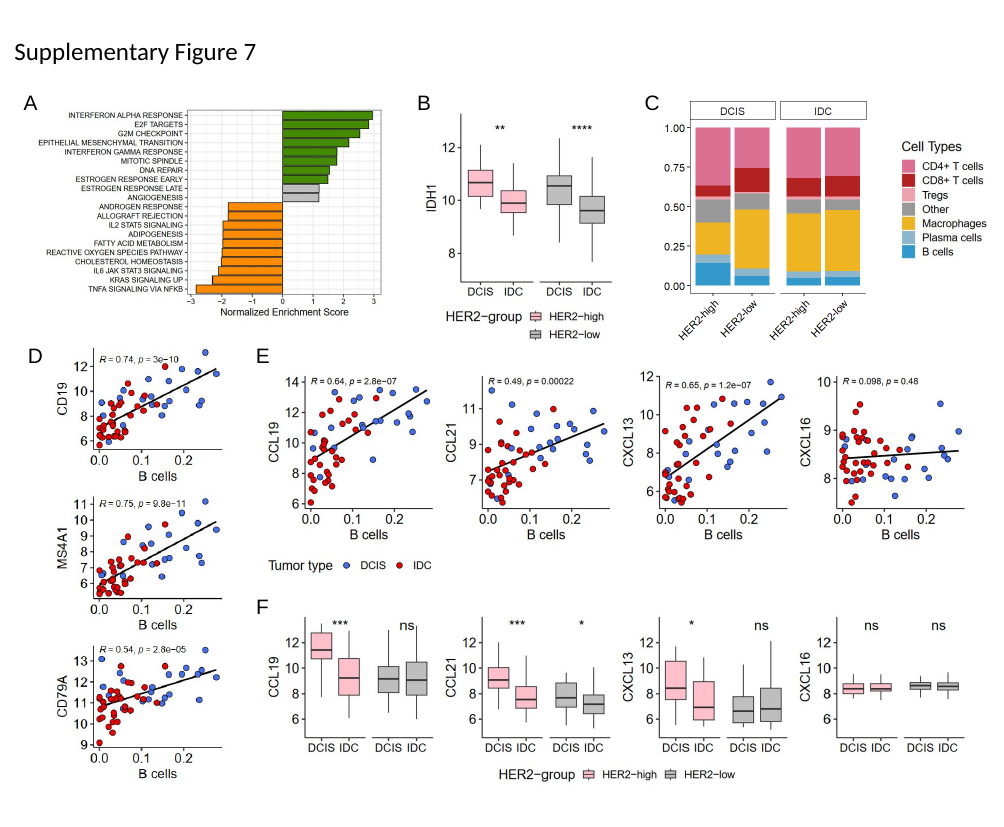

Supplementary Figure 7
A
B
C
D
E
F

## Slide 9
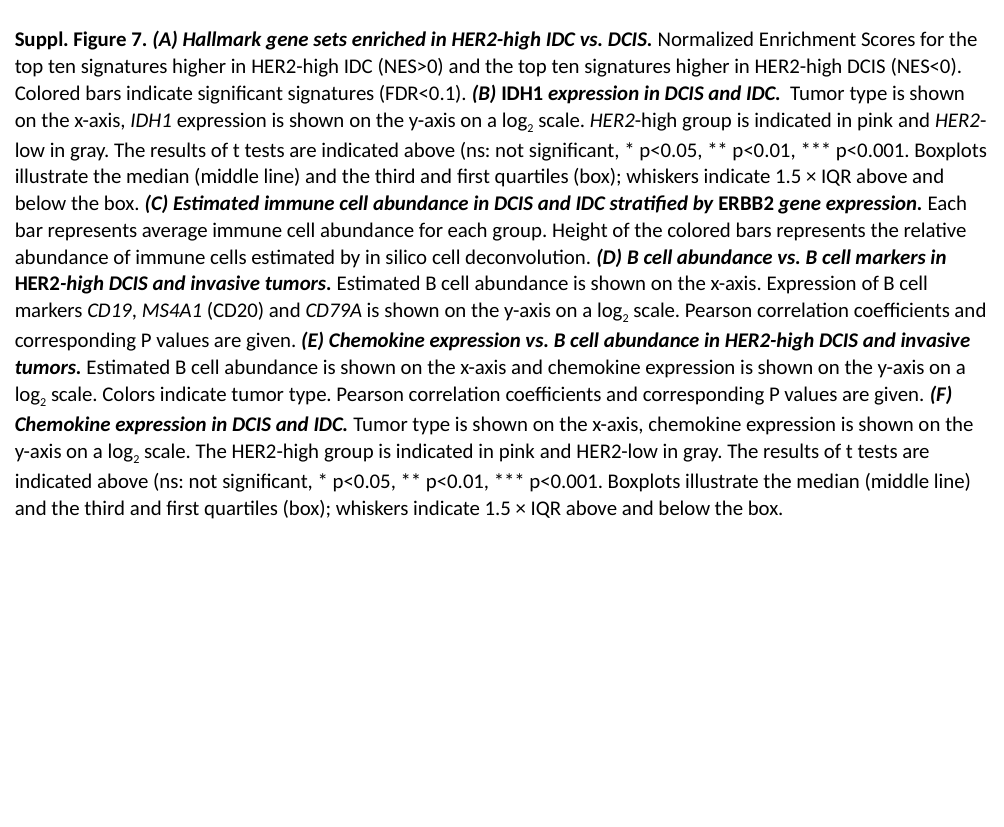

Suppl. Figure 7. (A) Hallmark gene sets enriched in HER2-high IDC vs. DCIS. Normalized Enrichment Scores for the top ten signatures higher in HER2-high IDC (NES>0) and the top ten signatures higher in HER2-high DCIS (NES<0). Colored bars indicate significant signatures (FDR<0.1). (B) IDH1 expression in DCIS and IDC. Tumor type is shown on the x-axis, IDH1 expression is shown on the y-axis on a log2 scale. HER2-high group is indicated in pink and HER2-low in gray. The results of t tests are indicated above (ns: not significant, * p<0.05, ** p<0.01, *** p<0.001. Boxplots illustrate the median (middle line) and the third and first quartiles (box); whiskers indicate 1.5 × IQR above and below the box. (C) Estimated immune cell abundance in DCIS and IDC stratified by ERBB2 gene expression. Each bar represents average immune cell abundance for each group. Height of the colored bars represents the relative abundance of immune cells estimated by in silico cell deconvolution. (D) B cell abundance vs. B cell markers in HER2-high DCIS and invasive tumors. Estimated B cell abundance is shown on the x-axis. Expression of B cell markers CD19, MS4A1 (CD20) and CD79A is shown on the y-axis on a log2 scale. Pearson correlation coefficients and corresponding P values are given. (E) Chemokine expression vs. B cell abundance in HER2-high DCIS and invasive tumors. Estimated B cell abundance is shown on the x-axis and chemokine expression is shown on the y-axis on a log2 scale. Colors indicate tumor type. Pearson correlation coefficients and corresponding P values are given. (F) Chemokine expression in DCIS and IDC. Tumor type is shown on the x-axis, chemokine expression is shown on the y-axis on a log2 scale. The HER2-high group is indicated in pink and HER2-low in gray. The results of t tests are indicated above (ns: not significant, * p<0.05, ** p<0.01, *** p<0.001. Boxplots illustrate the median (middle line) and the third and first quartiles (box); whiskers indicate 1.5 × IQR above and below the box.
